# The distinct role of CD4^+^ and CD8^+^ T-cells during the anti-tumour effects of targeted superantigens

**DOI:** 10.1038/sj.bjc.6690701

**Published:** 1999-09

**Authors:** M J Litton, M Dohlsten, A Rosendahl, L Ohlsson, M Søgaard, J Andersson, U Andersson

**Affiliations:** Department of Immunology, Wenner-Gren's Institute, Stockholm University, Stockholm, S-106 91, Sweden; Active Biotech, PO Box 724, Lund, S-220 07, Sweden; Department of Tumor Immunology, Wallenberg Laboratory, University of Lund, Lund, Sweden; Department of Microbiology, Pathology and Infectious Diseases, Karolinska Institute, Huddinge Hospital, Stockholm, Sweden; Department of Pediatrics, Karolinska Institute, St Göran's Children's Hospital, Stockholm, Sweden; AstraZeneca, R&D Lund, Lund, S-221 87, Sweden

**Keywords:** tumour therapy, superantigen, cytokines, perforin, knock out mice, T-cells

## Abstract

To target T-cells to the tumour area we created a recombinant protein of the bacterial superantigen (SAg) Staphylococcal enterotoxin A (SEA) and the Fab-fragment of a tumour-reactive antibody. This antibody-targeted SAg immunotherapy therapy has been shown to be highly efficient, eliminating > 95% of the pulmonary metastasis in mice carrying established melanoma micrometastases. Earlier studies demonstrated that elimination of the C215-expressing B16-melanoma lung metastasis was dependent on interferon (IFN)-γ release and expression of perforin. In the present study, therapeutic effector functions were analysed both locally at the tumour site and systemically in the spleen. In order to elucidate the role of each T-cell subset during Fab–SEA therapy, CD4 knock-out (KO) and CD8 KO mice were used. Tumour size reduction was statistically significant in Fab–SEA-based tumour therapy in both types of T-cell-deficient mice compared to wild-type mice. CD4 KO mice displayed a drastic reduction in the number of tumour-infiltrating macrophages and CD8^+^ T-cells. Therapy-induced accumulation of perforin-containing cells at the tumour site was significantly impaired in CD8 KO mice, and marginally in CD4 KO mice. Moreover, CD4 KO mice failed to produce substantial amounts of the tumour suppressive cytokine IFN-γ. This is in sharp contrast to normal mice where a massive local release was recorded. CD8 KO mice displayed a spontaneous production of interleukin (IL)-4 and IL-10 locally in the tumour. Neither normal nor CD4 KO mice produced detectable levels of these Th-2-associated cytokines. The high level of IL-10 was demonstrated to inhibit Fab–SEA tumour therapy, since the therapeutic efficacy was significantly higher in IL-10 KO mice. These results illustrate the importance of a finely tuned cellular collaboration to regulate the various phases of an efficient anti-tumour immune response. © 1999 Cancer Research Campaign


*British Journal of Cancer* (1999) **81**(2), 359–366
© 1999 Cancer Research Campaign
Article no. bjoc.1999.0701

### The distinct role of CD4+ and CD8+ T-cells during the anti-tumour effects of targeted superantigens

**MJ Litton[1], M Dohlsten[3,6], A Rosendahl[2,6], L Ohlsson[2], M Søgaard[2], J Andersson[1,4] and U Andersson[1,5]**

[1]Department of Immunology, Wenner-Gren's Institute, Stockholm University, S-106 91 Stockholm, Sweden; [2]Active Biotech, PO Box 724, S-220 07 Lund, Sweden; [3]Department of Tumor Immunology, Wallenberg Laboratory, University of Lund, Lund, Sweden; [4]Karolinska Institute, Department of Microbiology, Pathology and Infectious Diseases, Huddinge Hospital, Stockholm, Sweden; [5]Karolinska Institute, Department of Pediatrics, St Göran's Children's Hospital, Stockholm, Sweden; [6]AstraZeneca, R&D Lund, S-221 87 Lund, Sweden

**Summary** To target T-cells to the tumour area we created a recombinant protein of the bacterial superantigen (SAg) Staphylococcal enterotoxin A (SEA) and the Fab-fragment of a tumour-reactive antibody. This antibody-targeted SAg immunotherapy therapy has been shown to be highly efficient, eliminating > 95% of the pulmonary metastasis in mice carrying established melanoma micrometastases. Earlier studies demonstrated that elimination of the C215-expressing B16-melanoma lung metastasis was dependent on interferon (IFN)-γ release and expression of perforin. In the present study, therapeutic effector functions were analysed both locally at the tumour site and systemically in the spleen. In order to elucidate the role of each T-cell subset during Fab–SEA therapy, CD4 knock-out (KO) and CD8 KO mice were used. Tumour size reduction was statistically significant in Fab–SEA-based tumour therapy in both types of T-cell-deficient mice compared to wild-type mice. CD4 KO mice displayed a drastic reduction in the number of tumour-infiltrating macrophages and CD8+ T-cells. Therapy-induced accumulation of perforin-containing cells at the tumour site was significantly impaired in CD8 KO mice, and marginally in CD4 KO mice. Moreover, CD4 KO mice failed to produce substantial amounts of the tumour suppressive cytokine IFN-γ. This is in sharp contrast to normal mice where a massive local release was recorded. CD8 KO mice displayed a spontaneous production of interleukin (IL)-4 and IL-10 locally in the tumour. Neither normal nor CD4 KO mice produced detectable levels of these Th-2-associated cytokines. The high level of IL-10 was demonstrated to inhibit Fab–SEA tumour therapy, since the therapeutic efficacy was significantly higher in IL-10 KO mice. These results illustrate the importance of a finely tuned cellular collaboration to regulate the various phases of an efficient anti-tumour immune response.

© 1999 Cancer Research Campaign

**Keywords:** tumour therapy; superantigen; cytokines; perforin; knock out mice; T-cells

A successful anti-tumour immune response requires an activation of several distinct but interacting immune effector functions resulting in a localized cytotoxic attack. Effector T-cells have been demonstrated in both human and animal studies to have the capacity of mediating immune responses against certain tumours (Rosenberg et al, 1985; Klarnet et al, 1989; Kahn et al, 1991; Hom et al, 1991). In the murine immune system CD4+ helper T-cells can be distinguished into either 'Th-1' cells that facilitate cell-mediated cytotoxicity by the production of tumour necrosis factor (TNF)-α, interlukin (IL)-2 and interferon (IFN)-α, or into 'Th-2' cells that stimulate B-cell growth, differentiation and antibody production by the secretion of IL-4, IL-5, IL-6 and IL-10 (Mosmann et al, 1989). IL-4 and IL-10 may also act as immunoregulatory cytokines since they inhibit a number of Th-1 effector functions and may block overall macrophage cytotoxicity (Oswald et al, 1992; Sundstedt et al, 1997). Moreover, IL-10 has been shown to suppress both IFN-γ production and the anti-tumour effector phase of IFN-γ (Aruga et al, 1997; Groux et al, 1998).

Superantigens (SAg) are bacterial and viral proteins that share the ability to activate a large number of T-cells as well as macrophages. Bacterial SAg bind to major histocompatibility complex (MHC) class II molecules as unprocessed proteins and subsequently interact with a high number of T-cells expressing particular T-cell receptor Vβ chains (White et al, 1989; Janeway et al, 1989). SAg activate both CD4+ and CD8+ T-cells and are very potent inducers of lymphokines and monokines, as well as of T-cell cytotoxicity (Langford et al, 1978; Andersson et al, 1992; Dohlsten et al, 1993; Scherer et al, 1993; Dhein et al, 1995). In order to enhance immunogenicity of tumour cells we have genetically fused the Fab region of a tumour-reactive monoclonal antibody (mAb) with the SAg Staphylococcal enterotoxin A (Fab–SEA) (Dohlsten et al, 1994, 1995).

Poorly immunogenic B16 melanoma cells were transfected with the gene encoding for human colon carcinoma antigen C215 and used to evaluate the effects of C215 Fab–SEA treatment in a syngeneic lung metastasis model (Dohlsten et al, 1995; Rosendahl et al, 1996). Treatment with C215 Fab–SEA fusion protein eradicated 95% of pulmonary B16–C215 melanoma metastasis in fully immunocompetent mice (Dohlsten et al, 1995; Rosendahl et al, 1996).

Recently, the systemic and local immune responses after repeated injections of Fab–SEA were demonstrated to generate two distinct, but coupled, immune reactions (Litton et al, 1997). TNF-α, MIP-1α and MIP-1-β were immediately synthesized at the local tumour site in the lung tissue in response to the primary Fab–SEA injection. Concurrently, SEA-reactive T-cells produced high levels

*Received 28 October 1998*
*Revised 16 February 1999*
*Accepted 17 February 1999*

*Correspondence to:* Dr A Rosendahl, AstraZeneca, R&D Lund, S-221 87 Lund, Sweden of IL-2 in the spleen and began to proliferate. The primed and expanded SEA-reactive $CD4^+$ and $CD8^+$ T-cells accumulated in the tumour area in response to repeated Fab–SEA injections and produced large amounts of the tumour suppressive cytokines IFN-γ and TNF-α. Marginal production of IL-10 was detected in the tumour, while partial production was recorded in the spleen.

In the present study, CD4, CD8 and IL-10 knock-out (KO) mice were used to characterize the importance of the T-cell subsets and the regulatory role of IL-10 during Fab–SEA anti-tumour responses, locally at the tumour site or systemically in the spleen. This study illustrated the functional significance of both $CD4^+$ and $CD8^+$ T-cells in mediating cellular infiltration, Th-1/Th-2 cytokine release and the induction of perforin.

### MATERIALS AND METHODS

### Animals

C57B1/6 mice were obtained from Brommice (Ry, Denmark) and kept under pathogen-free conditions. The mice were 8–12 weeks of age when used in this study. CD4 and CD8 KO mice were a kind gift from T Mak and were generated using homologous recombination (Fung Leung et al, 1991; Rahemtulla et al, 1991) and back-crossed to the C57B1/6 background. IL-10 KO mice were obtained from B&K Universal Ltd, UK.

### Reagents

The construction and expression of C215 Fab–SEA (Fab–SEA) were performed as previously described (Dohlsten et al, 1994). The fusion protein was expressed in *Escherichia coli* K-12 UL635 (ara-14, xyl-7, ΔompT, T4R) and purified on a protein G Sepharose column (Pharmacia LKB Biotechnology), and fractions containing Fab–SEA were passed through a PD-10 column (Pharmacia LKB Biotechnology).

### Antibodies mAbs directed against IFN-γ (XMG1.2), IL-4 (11B11), CD4, CD8, CD11b was purchased from PharMingen (San Diego, CA, USA). Anti-perforin mAb was purchased from Kamiya Biomedical Company (Tukwila, WA, USA). The IL-10-reactive mAb 16E3 was provided by Dr John Abrams DNAX Research Institute (CA, USA).

### In vivo tumour therapy

C215-transfected B16-melanoma cells ($1 \times 10^5$) in 0.2 ml phosphate-buffered saline (PBS) with 1% syngeneic mouse serum were inoculated intravenously (i.v.) into the tail vein. Treatment with one i.v. injection (0.05–50 μg) of Fab–SEA per day for 4 days was initiated at day 5. Mice were sacrificed at day 21 and lung metastases counted. To detect cellular infiltration or cytokine production, treatment was initiated at day 18 when large lung metastases were present. Staining for infiltration was performed at various time points after the injections as indicated in Figure legends.

### Immunohistochemistry

Cryopreserved tissue sections were stained with the saponin–formaldehyde procedure as previously reported (Litton et al, 1994). Briefly, the sections were cut and fixed with 2% formaldehyde in PBS for 20 min at 20°C. The sections were incubated overnight with anti-cytokine or anti-perforin mAb at 2 μg $ml^{-1}$ with 0.1% saponin. Sections were then incubated with biotinylated mouse absorbed rabbit anti-rat 1:500 (Vector Labs, Burlingame, CA, USA) for 30 min, followed by an incubation for 30 min with ExtraAvidin AP (1:2870) (Sigma). The substrate used, New Fuchsin Red (Dako), was filtered for 10 min. For detection of surface markers the above procedure was repeated, but without saponin. To test the specificity of the immunohistochemistry, relevant recombinant cytokines were used to block specific cytokine staining as previously described (Litton et al, 1997).

### Computer-aided image analysis

All of the cytokines detected and cellular infiltration and perforin secretion were analysed as previously reported (Litton et al, 1997). Briefly, each tissue was quantified using specific colour detection to measure the amount of immunoreactivity. These detection values were then used for assessing all cytokines, cell phenotypes and controls. The standard non-paired two-tailed Student's *t*-test was used to evaluate and compare the therapeutical observations and the immunological results obtained from image analysis.

### Tumour growth inhibition in vitro

C215-transfected B16 melanoma cells (1700 cells per well) were cultured in the presence of 5 μg IFN-γ and increasing amounts of IL-10. The cell viability was analysed in a MTT (3-(4,5-dimethylthiazal-2-yl)-2,5-diphenyl tetrazolium bromide) assay after 72 h according to a standard protocol (Rosendahl et al, 1998*b*).

### RESULTS

### Tumour therapy with C215 Fab–SEA in CD4- and CD8-deficient mice

We have earlier demonstrated that four daily injections (1 injection per day for 4 days) of Fab–SEA significantly ($P > 0.01$) reduced the number of B16-C215 melanoma lung metastases (Rosendahl et al, 1996). In order to dissect the role of $CD4^+$ and $CD8^+$ T-cells during the therapy, CD4 KO or CD8 KO mice carrying B16-C215 melanoma were treated with Fab–SEA. Repeated administration of Fab–SEA significantly reduced the number of B16–C215-transfected melanoma cells ($P < 0.05$) in immunocompetent animals (Figure 1). In contrast, both CD4 KO and CD8 KO mice had a significant impairment in anti-tumour therapy (Figure 1). Most interestingly, CD8 KO mice induced a stronger anti-tumour response after Fab–SEA therapy when compared to CD4 KO (Figure 1). These results demonstrate the both $CD4^+$ and $CD8^+$ T-cells are required to perform optimal Fab–SEA tumour therapy.

### Spontaneous Th-2 cytokine production prior to therapy in CD8 KO mice

In normal and CD4 KO mice no infiltration of IL-4- or IL-10-producing T-cells was detected prior to Fab–SEA treatment (Figure 2). In contrast, cells that spontaneously produced both IL-4 and IL-10 were observed in the tumour area in untreated CD8

 © 1999 Cancer Research Campaign

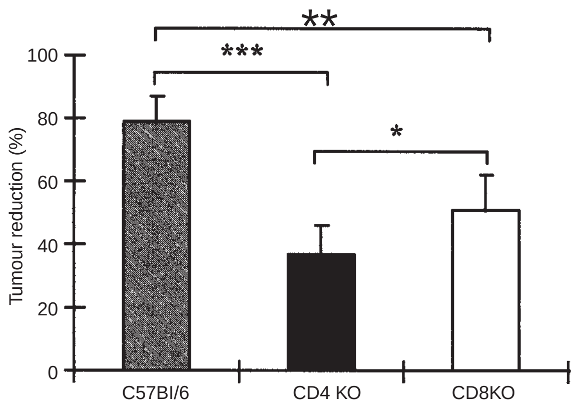

**Figure 1** Impaired Fab–SEA tumour therapy in CD4 and CD8 KO mice. The figure represents the reduction in B16–C215 tumour metastasis in normal C57B1/6, CD4 and CD8 KO mice. Animals were inoculated with $1 \times 10^5$ B16–C215 melanoma cells. On days 5–8, mice were treated i.v. with 50 µg of Fab–SEA. Lung metastases were counted on day 21. The number of lung metastases in untreated normal C57B1/6, CD4 and CD8 KO mice was 103, 108 and 77 respectively. Each group contained seven animals. Data are presented from one out of three similar experiments. The mean ± s.e.m. are shown. Statistical significance was analysed by Mann–Whitney *U*-test. *indicates $0.05 < P > 0.01$; **indicates $0.01 < P > 0.001$; ***indicates $P < 0.001$

KO mice (Figure 2AB). After repeated treatment with Fab–SEA, only a small number of IL-4- or IL-10-producing cells were detected in normal mice in spleen or in the lung (Figure 3A–D). In CD4 KO mice a marginal increase of IL-4- or IL-10-producing cells was recorded 24 h after the injection (Figure 3A–D). In sharp contrast, the frequency of IL-4- and IL-10-producing cells was two- to tenfold higher in CD8 KO compared to normal mice in both the tumour and spleen, but especially in the spleen (Figure 3A–D). These results suggest that $CD8^+$ T-cells may regulate the fine-tuning of cytokine production after Fab–SEA treatment.

### The functional importance of $CD4^+$ T-cells

We recently demonstrated that IFN-γ-producing tumour-infiltrating lymphocytes (TIL) infiltrate the tumour area in immunocompetent animals given four repeated injections of Fab–SEA (Dohlsten et al, 1995*a*). In order to determine which T-cell subset was required to obtain maximal infiltration of TIL, CD4 KO and CD8 KO mice were treated with Fab–SEA, and the cell infiltration as well as the IFN-γ production was monitored. Local IFN-γ production in normal mice was detected 2 h after the fourth injection and maximal production was recorded after 4 h (Figure 4A). Even 24 h after the fourth injection massive IFN-γ production was detected (Figure 4A). Similar to normal mice, CD8 KO mice induced substantial IFN-γ production in response to the fourth injection (Figure 4A). In sharp contrast, only marginal infiltration of IFN-γ-producing cells was recorded in CD4 KO mice (Figures 4A and 5A,B). In addition, the number of tumour-infiltrating $CD8^+$ T-cells was significantly reduced in CD4 KO mice after repeated Fab–SEA injections (Figure 4D). In both CD8 KO and CD4 KO mice the macrophage infiltration was reduced threefold compared to normal mice (Figure 4B). These results suggest that the IFN-γ production induced by Fab–SEA tumour therapy and the induction of an inflammatory milieu is dependent on CD4-reactive infiltrating TIL.

### Perforin secretion is induced after repeated Fab–SEA therapy

We recently demonstrated that Fab–SEA-based therapy of B16-C215 melanoma requires release of perforin (Rosendahl et al, 1998*b*). In order to investigate if Fab–SEA induced local release of perforin, lung metastases and spleen were analysed by staining for perforin after repeated Fab–SEA injections. Both locally in the tumour area and in the spleen, perforin secretion peaked after the third injection of Fab–SEA (Figure 6 A,B). Importantly, after three injections of Fab–SEA, CD8 KO mice had a significant reduction in the number of perforin-secreting cells (Figure 6C). These results suggest that optimal therapy of tumours sensitive to perforin requires an intact CD8 compartment.

### Improved therapeutic efficacy in the absence of IL-10

We have recently demonstrated that elimination of B16-C215 melanoma is dependent on IFN-γ production (Rosendahl et al, 1998*b*). Since IL-10 has been demonstrated to inhibit the production and effector phase of IFN-γ both directly (Aruga et al, 1997; Sundstedt et al, 1997) and indirectly, by modulation of the expression of MHC class II and co-stimulatory molecules (de Waal

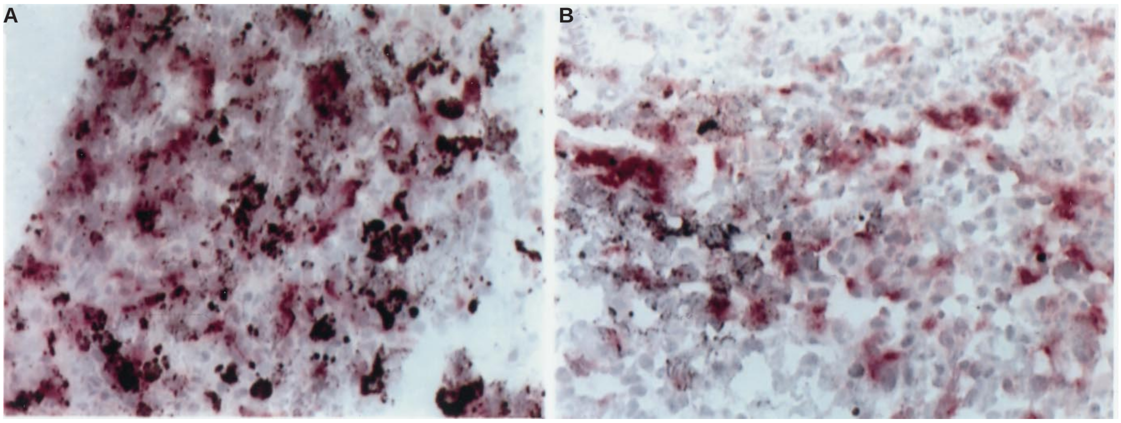

**Figure 2** Spontaneous Th-2 cytokine production prior to therapy in CD8 KO mice. The figure illustrates (**A**) IL-4- and (**B**) IL-10-producing cells stained by immunohistochemistry at the local tumour site prior to Fab–SEA therapy. Animals were treated with a single i.v. injection of 50 µg Fab–SEA on day 18 and sacrificed as indicated in figure. Data are presented from one out of two similar experiments

© 1999 Cancer Research Campaign 

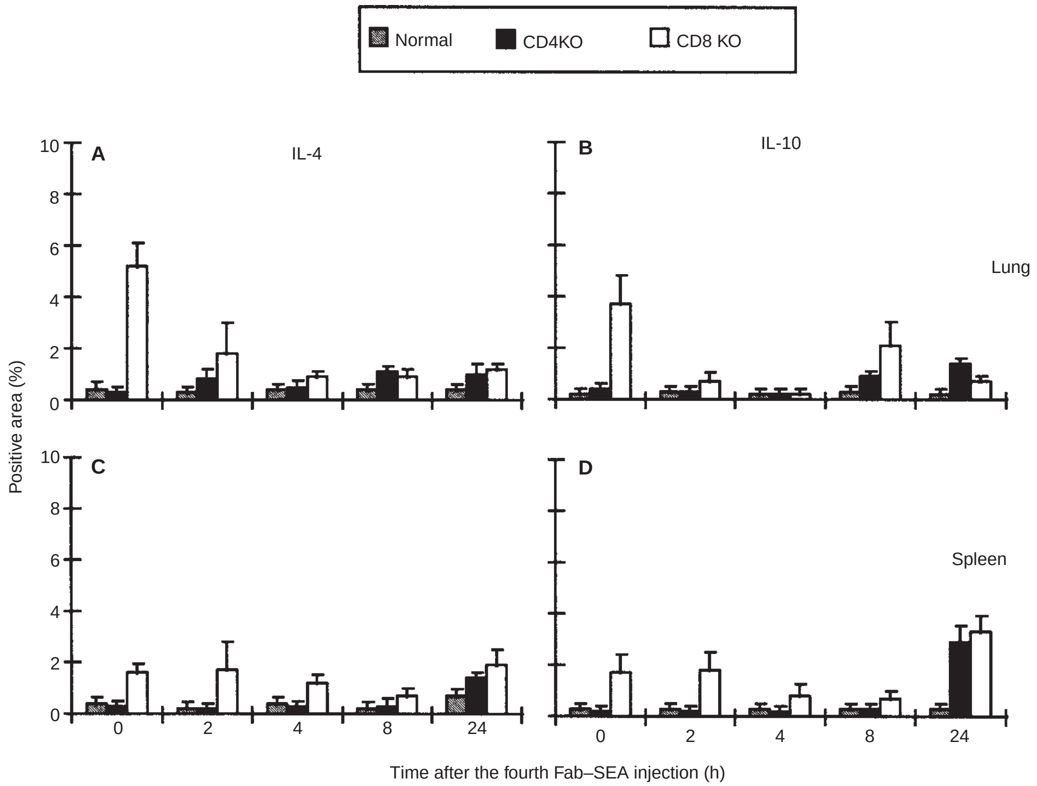

**Figure 3** Th-2 cytokine production locally in the tumour area. The production of IL-4 (**A, C**) and IL-10 (**B, D**) is quantified using computer-aided image analysis as a percentage of total lung (**A, B**) or spleen (**C, D**) tissue area. Animals were treated with four i.v. injections of 50 μg Fab–SEA on day 18–21 and sacrificed as indicated in figure. Data are presented from one out of two similar experiments. The mean ± s.e.m. are shown

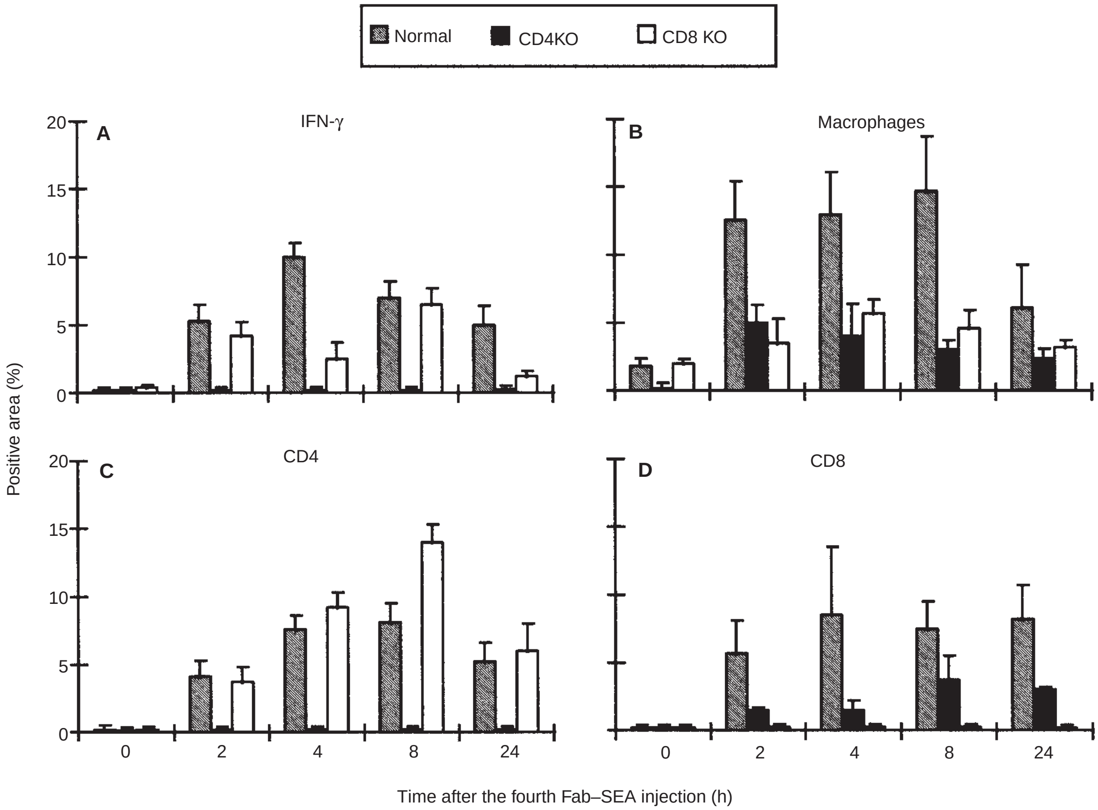

**Figure 4** Impaired infiltration of effector cells in CD4 KO mice. The figure illustrates the IFN-γ production (**A**), infiltration of macrophages (**B**), $CD4^+$ (**C**) and $CD8^+$ (**D**) quantified by computer-aided image analysis as a percentage of the total tumour lung tissue area. Animals were treated with four i.v. injections of 50 μg Fab–SEA on day 18–21 and sacrificed as indicated in the figure. Data are presented from one out of two similar experiments. The mean ± s.e.m. are shown

 © 1999 Cancer Research Campaign

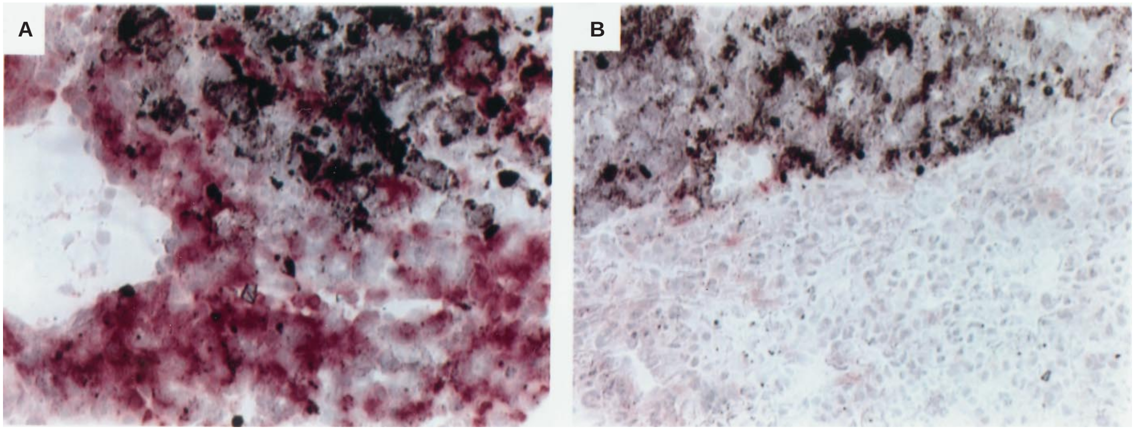

**Figure 5** The functional importance of CD4⁺ T-cells. The figure illustrates cellular production of IFN-γ stained by immunohistochemistry in normal C57B1/6 mice (**A**) or in CD4 KO mice (**B**). Animals were treated with four i.v. injections of 50 μg Fab–SEA on day 18–21 and sacrificed as indicated in figure. Data are presented from one out of two similar experiments

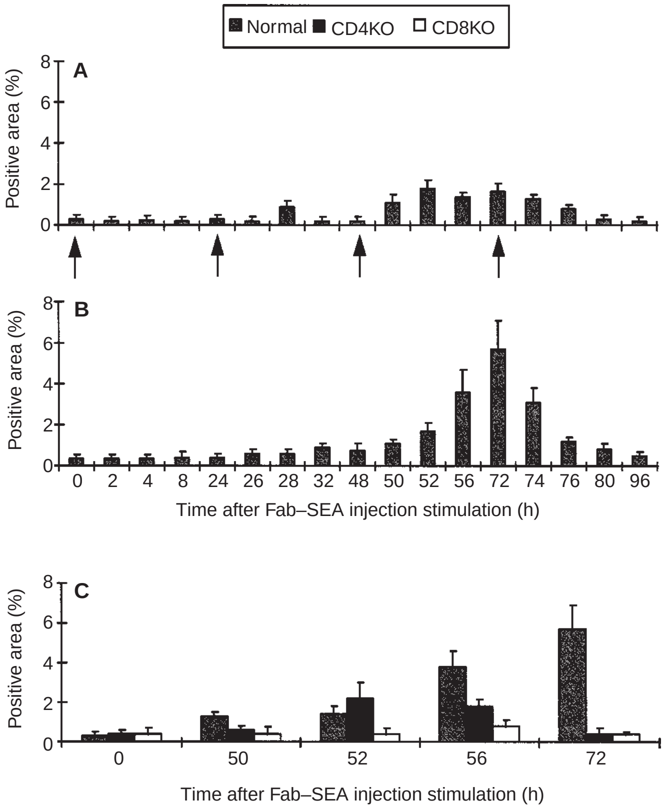

**Figure 6** Perforin expression is induced after Fab–SEA therapy. The figure illustrates perforin expression in C57B1/6 mice stained by immunohistochemistry in the spleen (**A**) and lung (**B**) quantified using computer-aided image analysis as a percentage of the total tissue area. Local perforin expression was compared in CD4 or CD8 KO mice versus C57B1/6 mice in the tumour lung tissue during the peak of secretion (**C**). Animals were treated with 1–4 i.v. injections of 50 μg Fab–SEA on day 18–21, as indicated by arrows. Data are presented from one out of two similar experiments. The mean ± s.e.m. are shown

Malefyt et al, 1991*a*, 1991*b*, Willems et al, 1994) we investigated if the therapeutic efficacy could be improved in IL-10 KO mice. When C215-transfected B16 melanoma cells were cultured in vitro in the presence of IFN-γ alone, marked tumour growth inhibition was recorded (Rosendahl et al, 1998*b*). In contrast, when IFN-γ and IL-10 both were present in culture, partial recovery of the B16-melanoma cell growth was noted, indicating that IL-10 modulated the growth inhibitory effects exhibited by IFN-γ (Figure 7A). Following treatment of B16-C215 lung metastasis with four daily injections of Fab–SEA, strong anti-tumour effects ($P > 0.01$) were seen in the IL-10 KO mice at doses from 0.05 μg to 50 μg (Figure 7B). Most importantly, at low doses (0.05–0.5 μg Fab–SEA per injection) significantly improved therapy ($P > 0.05$) was detected in IL-10 KO mice compared to wild-type mice

*© 1999 Cancer Research Campaign*

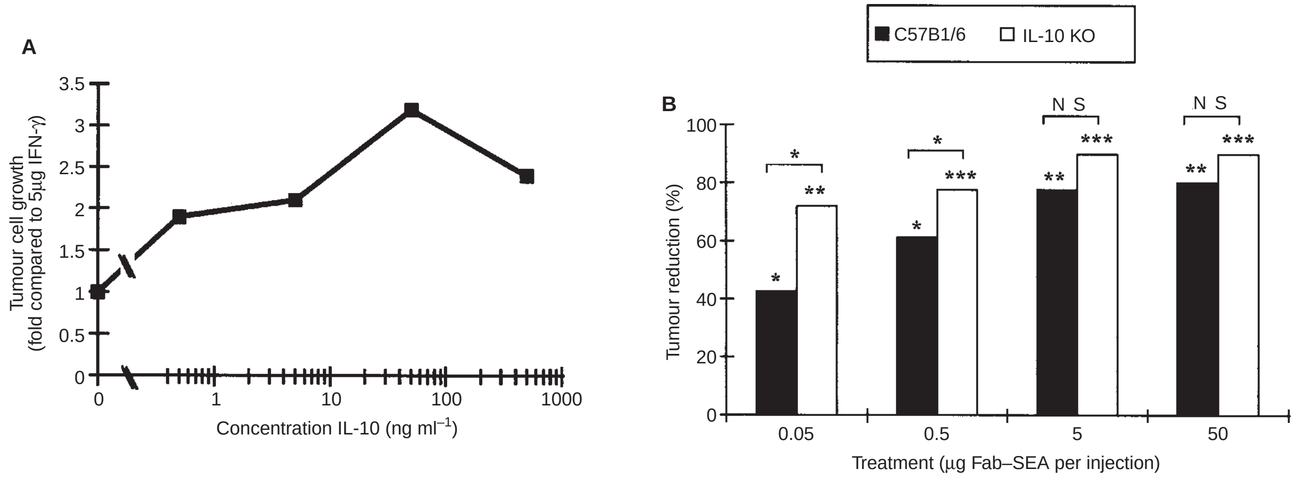

**Figure 7** IL-10 counteracts the IFN-γ-induced growth inhibition of B16-melanoma. C215-transfected B16 melanoma cells (1.7 × 10$^3$) were cultured in the presence of IFN-γ (5 ng ml$^{-1}$) or the combination of IFN-γ (5 ng ml$^{-1}$) and increasing amounts of IL-10 in vitro. After 72 h the viability was detected in MTT-assay. The values indicate a mean from a triplicate. One representative experiment out of three. (**C**) Animals were inoculated with 1 × 10$^5$ C215-transfected B16 melanoma cells. At day 4–7 the mice were treated with 50 µg Fab–SEA i.v. Lung metastases were counted at day 21. Each group contained seven animals. One out of two similar experiments. The significance was analysed by the Mann–Whitney *U*-test. *0.05 < *P* > 0.01; **0.01< *P* > 0.001; ****P* < 0.001. The mean ± s.e.m. are shown

(Figure 7B). This suggests that presence of IL-10 locally in the tumour area or in systemic blood limits the therapeutic efficacy of SAg–mAb fusion protein-based therapy.

### DISCUSSION

Mature T-cells expressing the T-cell receptor αβ heterodimer can be divided into two major subsets based on their exclusive expression of CD4 or CD8 surface molecules; these subsets of cells will bind to antigens presented in the context of MHC class II or class I molecules respectively. SAg have, in contrast to conventional peptide antigens, a unique capacity of activating both subsets of T-cells (Herrmann et al, 1990; Dohlsten et al, 1990). In fact, it has been demonstrated previously that both CD4$^+$ and CD8$^+$ T-cells are recruited to the tumour area in response to antibody-targeted SAg tumour therapy (Dohlsten et al., 1995*a*; Litton et al, 1996, 1997; Rosendahl et al, 1998*a*). However, both subsets have the potential to regulate helper activity as well as cytotoxic functions. It is therefore essential to investigate effector mechanisms by each subset during the course of Fab–SEA activation.

Similar to previous studies, repeated administration of Fab–SEA, given daily for 4 consecutive days, significantly (*P* < 0.01) reduced the number of B16–C215 pulmonary melanoma metastasis in immunocompetent animals when compared to untreated animals (Rosendahl et al, 1996). It was discovered that mice that lacked CD4$^+$ or CD8$^+$ T-cells had a significantly impaired therapeutic response. The results showed that CD8 KO mice mounted a stronger anti-tumour response after Fab–SEA therapy than CD4 KO mice. These results are in line with the previous findings that Fab–SEA therapy was unsuccessful in severe combined immune deficient (SCID) mice and nude mice, deficient of T-cells (Litton et al, 1996). Thus, T-cells are important mediators in SAg-targeted therapy. However, in the current study, neither CD4$^+$ nor CD8+ T-cells alone were able to effectively eradicate the tumours. This strongly suggests that the T-cells subsets collaborate to induce the appropriate anti-tumour response. Similar findings have been reported previously in other studies with murine and human tumours, but the underlying mechanisms have not been well characterized (Kern et al, 1986).

Spontaneous cytokine production at the local tumour site prior to Fab–SEA therapy was observed in CD8 KO mice, but not in CD4 KO or normal C57B1/6 mice. CD8$^+$ KO mice expressed cells that produced IL-4 and IL-10 in areas that were in close contact with the pulmonary tumours and in numerous cells in the spleen. These cytokine-producing cells were not detected prior to therapy in the other investigated animals. These results suggest that CD8$^+$ T-cells have the ability to alter the cytokine production of CD4$^+$ T-cells both at the local tumour site and the lymphoid organs. A possible candidate cell for the IL-4 synthesis could be CD4$^+$ NK1.1$^+$ T-cells that are known to produce IL-4 (Yoshimoto et al, 1994). The tendency of CD8 KO mice to produce Th-2 cytokines in the tumour vicinity remained after Fab-SEA therapy. In contrast, in normal C57B1/6 and CD4 KO mice IL-4- and IL-10-producing cells were only detected in the spleen after four injections of Fab–SEA, and were never detectable at the tumour site. These results combined with the reduced anti-tumour response would imply that in CD8 KO mice the tumour therapy was impaired due to the release of regulatory Th-2-type cytokines. This pattern is also seen during helminth infections where early IL-4 drives the response towards a Th-2-type response (Sher et al, 1992; Yoshimoto et al, 1994). IL-10 has been shown to down-regulate T-cell immune responses by various mechanisms. It may inhibit and down-regulate monocyte MHC class II expression as well as interfere with antigen presentation (Fiorentino et al, 1991), inhibit the up-regulation of co-stimulatory molecules (Ding et al, 1993) and suppress the cytotoxicity of macrophages (Giovarelli et al, 1995). In addition, it was recently demonstrated that T-cell receptor triggering in the presence of IL-10 inhibited IFN-γ production in freshly isolated CD4+ T-cells (Groux et al, 1996) and down-regulated the expression of granzyme B (Fitzpatrick et al, 1996). In vivo neutralization of IL-10 after SEA stimulation strongly augmented the production of the tumouricidal cytokines

 © 1999 Cancer Research Campaign

IFN-γ and TNF-α (Sundstedt et al, 1997). Moreover, Aruga et al (1997) demonstrated that in vivo neutralization of IL-10 strongly improved the anti-tumour response. In line with these experiments, we recorded elevated and prolonged IFN-γ production in IL-10 KO mice (data not shown). More importantly, significantly improved therapeutic efficacy was noted in IL-10 KO mice compared to normal mice. Thus, the low level of IL-10 induced after repeated stimulation with Fab–SEA seems to interfere with targeted SAg anti-tumour therapy in normal mice. It is tempting to speculate that the markedly elevated IL-10 levels recorded in CD8 KO mice in the tumour vicinity may in part account for the reduced therapeutic efficacy. Whether this is related to reduced expression of granzyme B remains to be investigated.

After four injections of Fab–SEA therapy the tumour infiltration of macrophages, $CD4^+$ and $CD8^+$ T-cells was markedly increased in immunocompetent mice. In contrast, the number of tumour-infiltrating macrophages was significantly reduced in both CD4 and CD8 KO mice after repeated Fab–SEA injections. In addition, mice deficient for $CD4^+$ T-cells had a severe reduction in the number of $CD8^+$ TIL compared to immunocompetent mice. These results imply that $CD4^+$ T-cells are necessary for the recruitment of $CD8^+$ TIL after therapy. This is in accordance with what others have found in allograft transplant rejection studies where mice that were deficient in $CD4^+$ T-cells were unable to reject transplants, even though these knockout mice had cytotoxic capabilities (Krieger et al, 1996). In other settings, $CD4^+$ T-cells have been shown to be essential in preventing the exhaustion of $CD8^+$ effector T-cells during a high viral load of lymphocytic choriomeningitis virus (LCMV) (Battegay et al, 1994). In line with these experiments, we recently demonstrated augmented $CD8^+$ proliferation, IFN-γ production, CTL activity and tumour cell eridication when Fab–IL-2 was injected in conjunction with Fab–SEA (Rosendahl et al, 1999). These results suggest that the anergic $CD4^+$ T-cells fail to produce IL-2 in levels required to activate the responsive 'memory' $CD8^+$ T-cells. Thus as Lee et al (1992) demonstrated, SAg fail to induce $CD4^+$ memory, while responsive $CD8^+$ T-cells' memory cells are induced (Coppola et al, 1997). It is tempting to speculate that the anergic $CD4^+$ T-cells fail to deliver activating signals to the $CD8^+$ T-cells, which then fail to perform anti-tumour effector mechanisms.

It was also demonstrated in the present study that CD4 KO mice were unable to produce IFN-γ, while CD8 KO mice and normal C57B1/6 mice generated significant numbers of IFN-γ-producing cells in response to therapy. This result, in combination with the lack of effective therapy observed in these mice, suggests that IFN-γ is an important mediator in the overall cytostatic anti-tumour response. Macrophages are known to mediate strong tumouricidal effects and depend on IFN-γ as a priming factor for tumour cytotoxicity (Pace et al, 1981; Schreiber, 1984). In addition, we recently demonstrated that therapy of the B16 melanoma strongly depends on release of IFN-γ (Rosendahl et al, 1998*b*). Therefore, IFN-γ probably exerts both direct and indirect anti-tumour effects.

A primary pathway for T-cell-mediated cytotoxicity is through perforin granular exocytosis. It has been illustrated that perforin KO mice have impaired cytotoxicity of CTL and natural killer cells (Kagi et al, 1994; Rosendahl et al, 1998*b*). In addition, we recently demonstrated that Fab–SEA-based therapy of B16-C215 melanoma is compromised in perforin KO mice (Rosendahl et al, 1998*b*). We now demonstrate that Fab–SEA-treated normal C57B1/6 mice display perforin secretion in the vicinity of tumour cells. In contrast, perforin secretion was strongly reduced in CD8 KO mice, and moderately in CD4 KO mice. This strengthens the notion that perforin secretion is important in the tumour eradication process and that this is dependent on an appropriate $CD4^+$ and $CD8^+$ T-cell interaction. It was illustrated in CD8 KO mice that the majority of perforin secretion was derived from $CD8^+$ T-cells. CD4 KO mice have an expanded population of $CD4^-CD8^-$T-cell receptor $\alpha/\beta^+$ (double negative) T-cells (Fung Leung et al, 1991) that possess suppressor characteristics (Schmidt Wolf et al, 1992). It is possible that this accounts for the low amount of perforin secretion in these animals. Since the number of tumour-infiltrating $CD8^+$ T-cells were reduced in mice lacking $CD4^+$ T-cells, it is difficult to determine if $CD4^+$ T-cells contribute to the induction of perforin or to the regulation of CTL infiltration.

The results in this study have demonstrated that $CD4^+$ T-cells provide help in recruiting $CD8^+$ T-cells and activating tumouricidal macrophages through IFN-γ production. $CD8^+$ T-cells initially suppressed a spontaneous prominent Th-2 response present in mice with pulmonary melanoma micrometastases, and then after Fab–SEA therapy became perforin-secreting effector cells at the local tumour site. Thus, during immunotherapy, both $CD4^+$ and $CD8^+$ T-cells collaborate to generate multiple anti-tumour effects to induce a maximized anti-tumour immune response.

### ACKNOWLEDGEMENTS

The skillful work of Ms Kristina Behm with the animal experiments is greatly acknowledged. The authors would like to acknowledge the gift of cytokine-specific antibodies from Drs. John Abrams (DNAX Research Institute, Palo Alto, CA), and Pharmingen (San Diego, CA). This work was supported by grants from the Swedish National Cancer Institute (grants #2490 and 2766), the Swedish Medical Research Council (grants #09082 and 10850), and von Kantzow's Foundation.

### REFERENCES

Andersson J, Nagy S, Bjork L, Abrams J, Holm S and Andersson U (1992) Bacterial toxin-induced cytokine production studied at the single-cell level. *Immunol Rev* **127**:

Aruga A, Aruga E, Tanigawa K, Bishop DK, Sondak VK and Chang AE (1997) Type 1 versus type 2 cytokine release by Vbeta T cell subpopulations determines in vivo antitumor reactivity: IL-10 mediates a suppressive role. *J Immunol* **159**: 664–673

Battegay M, Moskophidis D, Rahemtulla A, Hengartner H, Mak TW and Zinkernagel RM (1994) Enhanced establishment of a virus carrier state in adult CD4+ T-cell-deficient mice. *J Virol* **68**: 4700–4704

Coppola MA and Blackman MA (1997) Bacterial superantigens reactivate antigen-specific CD8+ memory T cells. *Int. Immunol* **9**: 1393–1403

de Waal Malefyt R, Abrams J, Bennett B, Figdor CG and de Vries JE (1991*a*) Interleukin 10 (IL-10) inhibits cytokine synthesis by human monocytes: an autoregulatory role of IL-10 produced by monocytes. *J Exp Med* **174**: 1209–1220

de Waal Malefyt R, Haanen J, Spits H, Roncarolo MG, te Velde A, Figdor C, Johnson K, Kastelein R, Yssel H and de Vries JE (1991*b*) Interleukin 10 (IL-10) and viral IL-10 strongly reduce antigen-specific human T cell proliferation by diminishing the antigen-presenting capacity of monocytes via downregulation of class II major histocompatibility complex expression. *J Exp Med* **174**: 915–924

Dhein J, Walczak H, Baumler C, Debatin KM and Krammer PH (1995) Autocrine T-cell suicide mediated by APO-1/(Fas/CD95). *Nature* **373**: 438–441

© 1999 Cancer Research Campaign 

Ding L, Linsley PS, Huang LY, Germain RN and Shevach EM (1993) IL-10 inhibits macrophage costimulatory activity by selectively inhibiting the up-regulation of B7 expression. *J Immunol* **151**: 1224–1234
Dohlsten M, Lando PA, Hedlund G, Trowsdale J and Kalland T (1990) Targeting of human cytotoxic T lymphocytes to MHC class II-expressing cells by staphylococcal enterotoxins. *Immunology* **71**: 96–100
Dohlsten M, Sundstedt A, Bjorklund M, Hedlund G and Kalland T (1993) Superantigen-induced cytokines suppress growth of human colon-carcinoma cells. *Int J Cancer* **54**: 482–488
Dohlsten M, Abrahmsen L, Bjork P, Lando PA, Hedlund G, Forsberg G, Brodin T, Gascoigne NR, Forberg C, Lind P, ET AL (1994) Monoclonal antibody-superantigen fusion proteins: tumor-specific agents for T-cell-based tumor therapy. *Proc Natl Acad Sci USA* **91**: 8945–8949
Dohlsten M, Hansson J, Ohlsson L, Litton M and Kalland T (1995*a*) Antibody-targeted superantigens are potent inducers of tumor-infiltrating T lymphocytes in vivo. *Proc Natl Acad Sci USA* **92**: 9791–9795
Dohlsten M, Lando PA, Bjork P, Abrahmsen L, Ohlsson L, Lind P and Kalland T (1995*b*) Immunotherapy of human colon cancer by antibody-targeted superantigens. *Cancer Immunol Immunother* **41**: 162–168
Fiorentino DF, Zlotnik A, Vieira P, Mosmann TR, Howard M, Moore KW and O'Garra A (1991) IL-10 acts on the antigen-presenting cell to inhibit cytokine production by Th1 cells. *J Immunol* **146**: 3444–3451
Fitzpatrick L, Makrigiannis AP, Kaiser M and Hoskin D (1996) Anti-CD3-activated killer T cells: interferon-gamma and interleukin-10 cross-regulate granzyme B expression and the induction of major histocompatibity complex-unrestricted cytotoxicity. *J Interferon Cytokine Res* **16**: 537–546
Fung Leung WP, Schilham MW, Rahemtulla A, Kundig TM, Vollenweider M, Potter J, van Ewijk W and Mak TW (1991) CD8 is needed for development of cytotoxic T cells but not helper T cells. *Cell* **65**: 443–449
Giovarelli M, Musiani P, Modesti A, Dellabona P, Casorati G, Allione A, Consalvo M, Cavallo F, di Pierro F, de Giovanni C, ET AL (1995) Local release of IL-10 by transfected mouse mammary adenocarcinoma cells does not suppress but enhances antitumor reaction and elicits a strong cytotoxic lymphocyte and antibody-dependent immune memory. *J Immunol* **155**: 3112–3123
Groux H, Bigler M, de Vries JE and Roncarolo MG (1996) Interleukin-10 induces a long-term antigen-specific anergic state in human CD4+ T cells. *J Exp Med* **184**: 19–29
Groux H, Bigler M, de Vries JE and Roncarolo MG (1998) Inhibitory and stimulatory effects of IL-10 on human CD8+ T cells. *J Immunol* **160**: 3188–3193
Herrmann T, Maryanski JL, Romero P, Fleischer B and MacDonald HR (1990) Activation of MHC class I-restricted CD8+ CTL by microbial T cell mitogens. Dependence upon MHC class II expression of the target cells and V beta usage of the responder T cells. *J Immunol* **144**: 1181–1186
Hom SS, Topalian SL, Simonis T, Mancini M and Rosenberg SA (1991) Common expression of melanoma tumor-associated antigens recognized by human tumor infiltrating lymphocytes: analysis by human lymphocyte antigen restriction. *J Immunother* **10**: 153–164
Janeway CA, Jr, Yagi J, Conrad PJ, Katz ME, Jones B, Vroegop S and Buxser S (1989) T-cell responses to Mls and to bacterial proteins that mimic its behavior. *Immunol Rev* **107**:
Kagi D, Ledermann B, Burki K, Hengartner H and Zinkernagel RM (1994) CD8+ T cell-mediated protection against an intracellular bacterium by perforin-dependent cytotoxicity. *Eur J Immunol* **24**: 3068–3072
Kahn M, Sugawara H, Mcgowan P, Okuno K, Nagoya S, Hellstrom KE, Hellstrom I and Greenberg P (1991) CD4+ T cell clones specific for the human p97 melanoma-associated antigen can eradicate pulmonary metastases from a murine tumor expressing the p97 antigen. *J Immunol* **146**: 3235–3241
Kern DE, Klarnet JP, Jensen MC and Greenberg PD (1986) Requirement for recognition of class II molecules and processed tumor antigen for optimal generation of syngeneic tumor-specific class I-restricted CTL. *J Immunol* **136**: 4303–4310
Klarnet JP, Kern DE, Okuno K, Holt C, Lilly F and Greenberg PD (1989) FBL-reactive CD8+ cytotoxic and CD4+ helper T lymphocytes recognize distinct Friend murine leukemia virus-encoded antigens. *J Exp Med* **169**: 457–467
Krieger NR, Yin DP and Garrison Fathman C (1996) CD4+ but not CD8+ cells are essential for allorejection. *J Exp Med* **184**: 2013–2018
Langford MP, Stanton GJ and Johnson HM (1978) Biological effects of staphylococcal enterotoxin A on human peripheral lymphocytes. *Infect Immun* **22**: 62–68
Lee WT and Vitetta ES (1992) Memory T cells are anergic to superantigen staphylococcal enterotoxin B. *J Exp Med* **176**: 575–579
Litton MJ, Sander B, Murphy E, O'Garra A and Abrams JS (1994) Early expression of cytokines in lymph nodes after treatment in vivo with Staphylococcus enterotoxin B. *J Immunol Methods* **175**: 47–58
Litton MJ, Dohlsten M, Lando PA, Kalland T, Ohlsson L, Andersson J and Andersson U (1996) Antibody-targeted superantigen therapy induces tumor-infiltrating lymphocytes, excessive cytokine production, and apoptosis in human colon carcinoma. *Eur J Immunol* **26**: 1–9
Litton MJ, Dohlsten M, Hansson J, Rosendahl A, Ohlsson L, Kalland T, Andersson J and Andersson U (1997) Tumor therapy with an antibody-targeted superantigen generates a dichotomy between local and systemic immune responses. *Am J Pathol* **150**: 1607–1618
Mosmann TR and Coffman RL (1989) TH-1 and TH-2 cells: different patterns of lymphokine secretion lead to different functional properties. *Annu Rev Immunol* **7**: 1967–1990
Oswald IP, Gazzinelli RT, Sher A and James SL (1992) IL-10 synergizes with IL-4 and transforming growth factor-β to inhibit macrophage cytotoxic activity. *J Immunol* **148**: 3578–3582
Pace JL and Russell SW (1981) Activation of mouse macrophages for tumor cell killing. I. Quantitative analysis of interactions between lymphokine and lipopolysaccharide. *J Immunol* **126**: 1863–1867
Rahemtulla A, Fung Leung WP, Schilham MW, Kundig TM, Sambhara SR, Narendran A, Arabian A, Wakeham A, Paige CJ, Zinkernagel RM, et al (1991) Normal development and function of CD8+ cells but markedly decreased helper cell activity in mice lacking CD4. *Nature* **353**: 180–184
Rosenberg SA, Lotze MT, Muul LM, Leitman S, Chang AE, Ettinghausen SE, Matory YL, Skibber JM, Shiloni E, Vetto JT, et al (1985) Observations on the systemic administration of autologous lymphokine-activated killer cells and recombinant interleukin-2 to patients with metastatic cancer. *N Engl J Med* **313**: 1485–1492
Rosendahl A, Hansson J, Sundstedt A, Kalland T and Dohlsten M (1996) Immune response during tumor therapy with antibody-superantigen fusion proteins. *Int J Cancer* **68**: 109–113
Rosendahl A, Kristensson K, Hansson J, Ohlsson L, Kalland T and Dohlsten M (1998*a*) Repeated treatment with antibody-targeted superantigens strongly inhibits tumor growth. *Int J Cancer* **76**: 274–283
Rosendahl A, Kristensson K, Hansson J, Riesbeck K, Kalland T and Dohlsten M (1998*b*) Perforin and IFN-gamma are involved in the antitumor effects of antibody-targeted superantigens. *J Immunol* **160**: 5309–5313
Scherer MT, Ignatowicz L, Winslow GM, Kappler JW and Marrack P (1993) Superantigens: bacterial and viral proteins that manipulate the immune system. *Annu Rev Cell Biol* **9**:
Schmidt Wolf IG, Dejbakhsh Jones S, Ginzton N, Greenberg P and Strober S (1992) T-cell subsets and suppressor cells in human bone marrow. *Blood* **80**: 3242–3250
Schreiber RD (1984) Identification of gamma-interferon as a murine macrophage-activating factor for tumor cytotoxicity. *Contemp Top Immunobiol* **13**:
Sher A, Gazzinelli RT, Oswald IP, Clerici M, Kullberg M, Pearce EJ, Berzofsky JA, Mosmann TR, James SL and Morse HC, III (1992) Role of T-cell derived cytokines in the downregulation of immune responses in parasitic and retroviral infection. *Immunol Rev* **127**: 183–204
Sundstedt A, Hoiden I, Rosendahl A, Kalland T, van Rooijen N and Dohlsten M (1997) Immunoregulatory role of IL-10 during superantigen-induced hyporesponsiveness in vivo. *J Immunol* **158**: 180–186
White J, Herman A, Pullen AM, Kubo R, Kappler JW and Marrack P (1989) The V beta-specific superantigen staphylococcal enterotoxin B: stimulation of mature T cells and clonal deletion in neonatal mice. *Cell* **56**: 27–35
Willems F, Marchant A, DelVille JP, Gerard C, Delvaux A, Velu T, de Boer M and Goldman M (1994) Interleukin-10 inhibits B7 and intercellular adhesion molecule-1 expression on human monocytes. *Eur J Immunol* **24**: 1007–1009
Yoshimoto T and Paul WE (1994) CD4pos, NK1.1pos T cells promptly produce interleukin 4 in response to in vivo challenge with anti-CD3. *J Exp Med* **179**: 1285–1295

 *© 1999 Cancer Research Campaign*
